# Supplementary material for: Neutralizing Antibody Response of the Wild-Type/Omicron BA.1 Bivalent Vaccine as the Second Booster Dose against Omicron BA.2 and BA.5
Source: Microbiol Spectr. 2023 Mar 22;11(2):e05131-22. doi: 10.1128/spectrum.05131-22 (PMC10101054; doi:10.1128/spectrum.05131-22)
Supplement: Supplemental file 1 — Supplemental material. Download spectrum.05131-22-s0001.pdf, PDF file, 0.6 MB [file spectrum.05131-22-s0001.pdf]

Supplemental Figures and Table:

Supplemental Figure 1. Anti-RBD antibody levels and neutralizing activity before the second booster dose

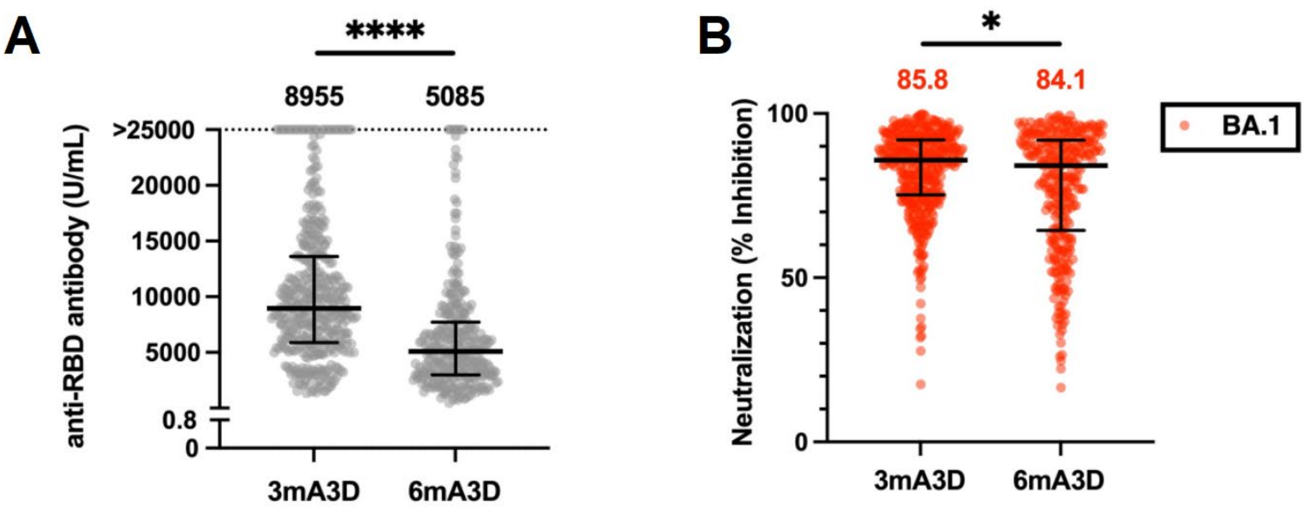

(A) Serum concentration of anti-RBD antibody at 3mA3D (n = 425) and 6mA3D (n = 321). Each dot represents an individual result. **(B)** Individual neutralizing activity against Omicron BA.1-pseudotyped virus at 3mA3D (n = 425) and 6mA3D (n = 321) using 100-fold diluted serum. The numbers at the top indicate the median neutralizing value for each group.

RBD, receptor-binding domain; 3mA3D, 3 months after the third dose; 6mA3D, 6 months after the third dose; *, $p < 0.05$; ****, $p < 0.0001$. Bars indicate medians with interquartile ranges.

Supplemental Figure 2. ID$_{50}$ titers against live Wild-type-, Omicron BA.1, BA.2, and BA.5 viruses at 2wA3D, 3mA3D, and 6mA3D

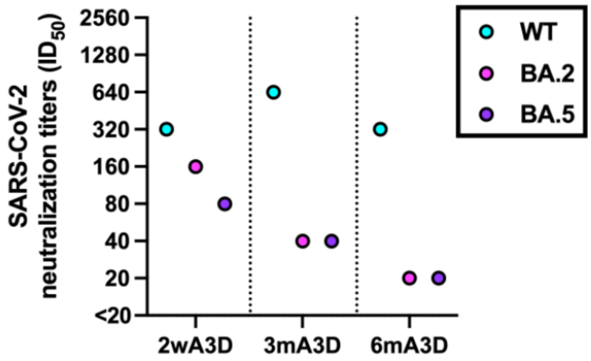

ID$_{50}$ titers against live SARS-CoV-2 viruses, WT, Omicron BA.2, and BA.5 at 2wA3D (n = 565), 3mA3D (n = 425), and 6mA3D (n = 321) using the pooled serum.

50% inhibitory dilution, ID$_{50}$; 2wA3D, 2 weeks after the third dose; 3mA3D, 3 months after the third dose; 6mA3D, 6 months after the third dose; 2wA4D, 2 weeks after the fourth dose.

Supplemental Figure 3. Relationship of vaccine-induced antibody levels and vaccine-related symptoms before the second booster dose in questionnaire-answered population

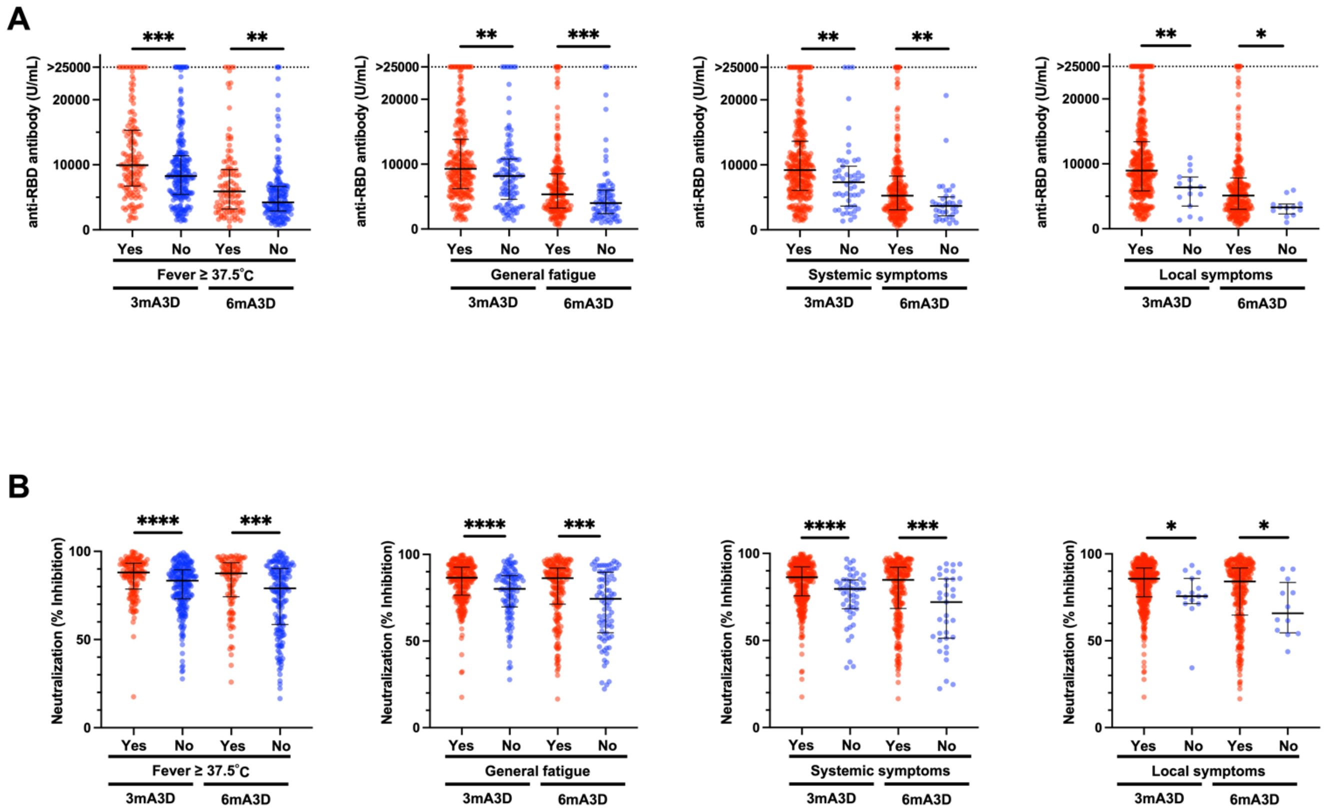

(A) Relationship between anti-RBD antibody levels and vaccine-related responses including fever ≥ 37.5 °C, general fatigue, systemic, and local symptoms at 3mA3D (n = 425) and 6mA3D (n = 321). **(B)** Relationship between neutralizing activity against the Omicron BA.1-pseudotyped virus and vaccine-related symptoms.

RBD, receptor-binding domain; 3mA3D, 3 months after the third dose; 6mA3D, 6 months

after the third dose; *, $p < 0.05$; **, $p < 0.01$; ***, $p < 0.001$; ****, $p < 0.0001$. Each dot represents an individual result. Bars indicate medians with interquartile ranges.

Supplemental Table 1. Relationship between vaccine-related symptoms after the first and second booster

Adverse reactions	WT group, n = 131				WT+BA.1 group, n = 22			
	First booster, n (%)		Second booster, n (%)		First booster, n (%)		Second booster, n (%)	
Fever ≥ 37.5 °C	Yes	44 (33.6)	Yes	30 (68.2)	Yes	12 (54.5)	Yes	8 (66.7)
			No	14 (31.8)			No	4 (33.3)
	No	87 (66.4)	Yes	24 (27.6)	No	10 (45.5)	Yes	1 (10.0)
			No	63 (72.4)			No	9 (90.0)
General fatigue	Yes	89 (67.9)	Yes	79 (88.8)	Yes	18 (81.8)	Yes	14 (77.8)
				10 (11.2)			No	4 (22.2)
	No	42 (32.1)	No	19 (45.2)	No	4 (18.2)	Yes	1 (25.0)
				23 (54.7)			No	3 (75.0)
At least one systemic symptom	Yes	109 (83.2)	Yes	100 (91.7)	Yes	20 (90.9)	Yes	17 (85.0)
			No	9 (8.3)			No	3 (15.0)
	No	22 (16.8)	Yes	9 (40.9)	No	2 (9.1)	Yes	1 (50.0)
			No	13 (59.1)			No	1 (5.0)
	Yes	124	Yes	120 (96.8)	Yes	22	Yes	22 (100.0)

At least		(94.7)	No	4 (3.2)		(100.0)	No	0 (0.0)
one local		7	Yes	5 (71.4)			Yes	-
	No				No	0 (0.0)		
symptom		(5.3)	No	2 (28.6)			No	-
